# Morris B. Bender (1905–1983)

**DOI:** 10.1007/s00415-025-13603-1

**Published:** 2026-01-08

**Authors:** Andrew J. Larner

**Affiliations:** Honorary Senior Research Fellow, Department of Translational Neuroscience & Stroke, Institute of Neurology, University College London, London, WC1E 6BT UK

The January–February 1974 issue of *The Mount Sinai Journal of Medicine* carried the title “Morris B. Bender: An Appreciation” and included his extensive Curriculum Vitae and Bibliography (some 17 pages, totalling 325 items). Few neurologists receive such an honour during their lifetime, let alone on their notional retirement (more publications followed, final count 367). However, only one paper amongst those included in this commemorative volume related to the condition which, in retrospect, was perhaps Bender’s most novel and enduring contribution to clinical neurology, namely his observation and description of what later came to be called “transient global amnesia”. Hence, this aspect of his work is emphasised here.

Born in Uman, Russia, Bender (Fig. 1) was brought to Philadelphia as a child. He qualified from the University of Pennsylvania (1931). In addition to his clinical training, he undertook laboratory work as Research Fellow in neurophysiology at Yale (1936–8) with John F. Fulton, noted physiologist and medical historian, co-authoring two papers with him (1938–9). However, Bender’s principal affiliation was to the Mount Sinai Hospital in New York, initially as a Resident (1933), eventually becoming Neurologist in Chief (1951) and Professor and Chairman (1966). He was also Head of the Laboratory of Experimental Neurology at New York University College of Medicine (1942–50). He was later the President of the American Neurological Association (1972–3) [1, 2].

**Fig. 1:**
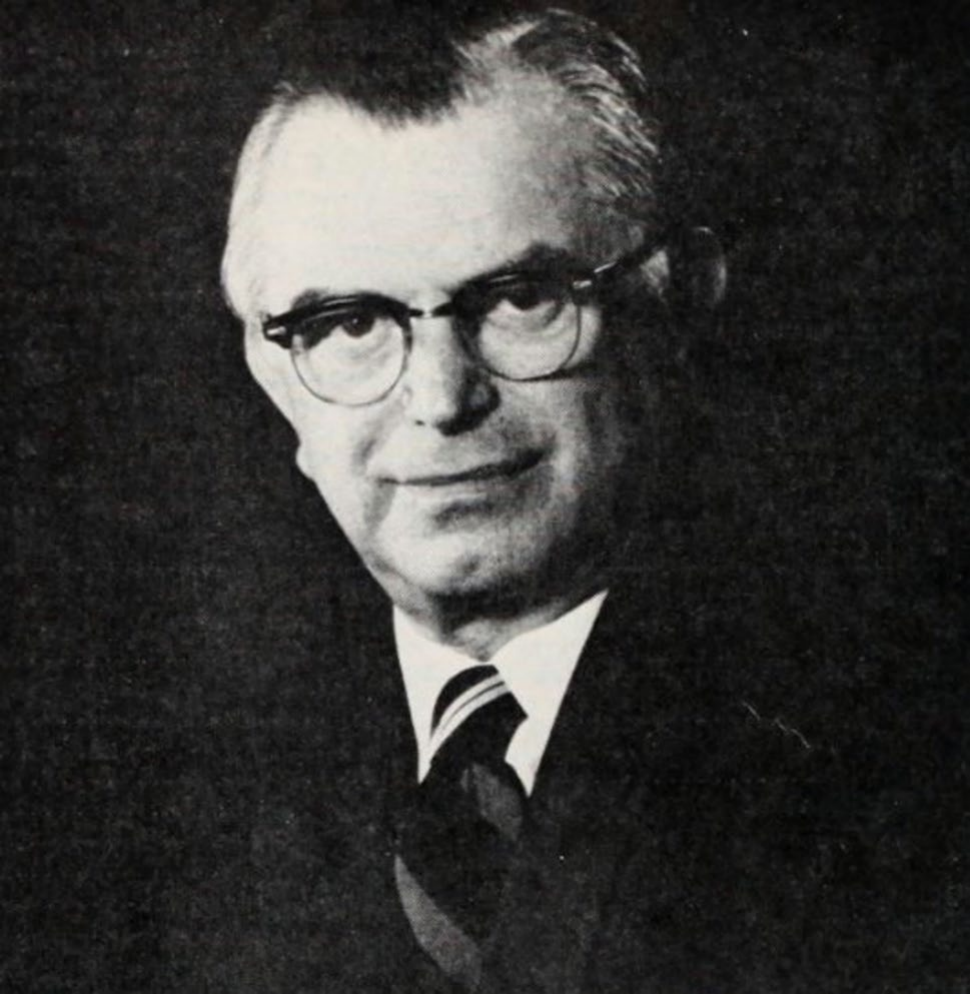
Morris B. Bender (1905–1983)

Two subjects dominate Bender’s published output: neuro-ophthalmology and perceptual function, both subjects of scrutiny in the 1974 commemorative volume [3, 4]. It was opined that “There is hardly an issue in neuro-ophthalmology to which Bender has not made significant clinical or experimental contributions” [3]. These included brainstem oculomotor pathways, particularly the lesion responsible for the medial longitudinal fasciculus syndrome, optokinetic nystagmus, and eye movements in states of altered consciousness including sleep, as well as phenomena such as palinopsia and oscillopsia.

Bender’s interest in sensory defects may have been initiated by exposure to injured soldiers with brain trauma at the US Naval Hospital in San Diego during the Second World War. His studies of visual and tactile disorders resulted in many papers and also a monograph which particularly recognised the value of double simultaneous sensory stimulation demonstrating the phenomenon of extinction [5]. If one might cherry-pick one item from his extensive oeuvre, his intriguing description of “exosomesthesia”, in which the patient mislocalizes sensation into the surrounding space [6], may perhaps have recapitulated an observation from the previous century by Charles Dickens who, in his novel *Hard Times* (1854), has a character locate her pain as “somewhere in the room”.

In addition to these subjects, Bender also wrote on cerebrovascular disease and aneurysms, and also the nonsurgical treatment of brain tumours and subdural haematoma. Indeed his *New York Times* obituary notice characterised him as an “expert on brain tumors”. His final paper, published posthumously, described neurosarcoidosis presenting as major depression (*J Neurol Neurosurg Psychiatry* 1984;47:1050-1).

In October 1956, Bender published on “Syndrome of isolated episode of confusion with amnesia”. This short paper described “12 post-menopausal patients suffering non-recurring episodes of defective memory with subsequent amnesia”, clinical phenomena which Bender adjudged to “defy current classification”. Episodes were characterised by a “single brief period of defective memory and confusion with a complete retrograde amnesia”. Repetition of the same questions by the patient was a frequent clinical observation. Attacks were reported to last for a few hours but did not recur. Bender’s favoured pathophysiological explanation was a “transient circulatory disturbance of the brain” [7].

In a subsequent paper, published 4 years later, Bender detailed the clinical features in 26 patients with a “Single episode of confusion with amnesia” and continued to emphasise the absence of recurrence since none had a second event recorded in up to 10 years of follow-up [8]. In the meantime, Fisher and Adams had reported their observations on “transient global amnesia” to the American Neurological Association (*Trans Am Neurol Assoc* 1958;83:143–146). Bender adopted this terminology, in addition to his own, in his final contribution to the subject, in which he reported on 51 patients, with particular reference to the normality of EEG findings as recorded in 27 of the cases, in 5 during the event itself [9]. By this time, Fisher and Adams’s monograph had appeared (*Acta Neurol Scand* 1964;40(Suppl9):1–81), definitively establishing the nomenclature of “transient global amnesia” (TGA).

Why are Bender’s contributions to TGA neglected compared to those of Fisher and Adams? Besides lacking a catchy nomenclature (even though the amnesia is not “global”, as the fractionated nature of memory function is now understood), Bender’s first paper had appeared in the *Journal of the Hillside Hospital* [7], a relatively obscure publication, house journal of a psychiatric facility in Glen Oaks, New York (Bender had no appointment there), so perhaps was read more by psychiatrists rather than neurologists. It may also relate to Bender’s insistence that the amnesic episodes were single, a position already controverted by another early (and neglected) account in the French literature by Guyotat and Courjon (“ictus amnésique”; *J Med Lyon* 1956;37:697–701) and also by Fisher and Adams in their 1964 monograph.

Bender initially suggested that “future biochemical studies may clarify the etiology” of TGA [7], a hope that has not materialised. However, another of his interests may have proved more applicable to elucidating the pathophysiology of TGA: the disciplines of control theory and cybernetics, pioneered in the 1940s and 1950s by mathematicians such as Norbert Wiener and John von Neumann. Bender attended cybernetics meetings (also known as the Macy Foundation meetings) on four occasions (dates not specified) [10]. These sought to apply concepts of feedback not only to mechanical and electrical engineering, chemistry, economics, and meteorology, but also to biology and human physiology. It was presumably Bender’s interest in eye movements which prompted his attendance, as a system potentially susceptible to modelling using feedback concepts [3]. Perhaps ironically, however, the characterisation of multiple loops embedded within the neuroanatomy of the hippocampal formation has more recently prompted ideas about the possible role of feedback mechanisms in hippocampal function and dysfunction, and specifically in the pathogenesis of TGA (*Cortex* 2022;149:137–147).

## Data Availability

Data sharing is not applicable to this article as no datasets were generated or analysed during the current study.
